# Age-Related Patterns in Trace Element Content Vary Between Bone and Teeth of the European Roe Deer (*Capreolus capreolus*)

**DOI:** 10.1007/s00244-017-0470-1

**Published:** 2017-10-25

**Authors:** Jan Demesko, Janusz Markowski, Mirosława Słaba, Janusz Hejduk, Piotr Minias

**Affiliations:** 1 Department of Biodiversity Studies and Bioeducation, Faculty of Biology and Environmental Protection, University of Łódź, Banacha 1/3, 90-237 Łódź, Poland; 2 Department of Industrial Microbiology and Biotechnology, Faculty of Biology and Environmental Protection, University of Łódź, Banacha 12/16, 90-237 Łódź, Poland

## Abstract

Game animals, such as the roe deer (*Capreolus capreolus*), have long been used as bioindicators of environmental contamination. Most ecotoxicological research on ungulates has focused on trace element content in soft tissues and antlers. Also, only fragmentary information exists about whether and how trace element concentrations vary with the age of wild-living animals and whether these age-related patterns are similar for different types of tissues. The purpose of this study was to measure concentrations of seven trace metals (barium, copper, iron, lead, manganese, strontium, zinc) and fluoride in bone and teeth of roe deer and to determine whether significant variation is evident with individual age. For this purpose, we collected permanent molars and fragments of mandible bone from more than 130 female roe deer in Central Poland. We found that concentrations of four trace elements (barium, manganese, zinc, and fluoride) in teeth of deer showed positive linear relationships with individual age. No such trends were recorded for trace element content in bone. We suggest that these striking differences in age-related patterns of trace element bioaccumulation between bone and permanent teeth of roe deer might be explained by higher turnover rate and constant remodelling of bone tissue. The results suggest that analysis of permanent teeth may be useful for assessing throughout-life intoxication by environmental pollution in the roe deer and possibly in other mammal species. Our study reinforces the need to carefully account for age-related variation in ecotoxicological research on wild-living animals.

**Electronic supplementary material:**

The online version of this article (doi:10.1007/s00244-017-0470-1) contains supplementary material, which is available to authorized users.

Technological developments and changes that occur in the modern world have a significant impact on natural environment. It is widely acknowledged that urbanization constitutes one of the most important threats to wildlife and its biodiversity in the present day (McKinney 2008). Expanding urban landscape, industrialization, and modern agriculture practices are all associated with growing emission of gaseous and particulate matter to the environment (Pacyna and Pacyna 2001; Pacyna et al. 2007; Norgate et al. 2007; Garcia et al. 2011). Contamination from burned fuel and other sources gets to the air, water, and plants (Markert 1993; Kabata-Pendias and Pendias 2001; Palczewska-Komsa et al. 2016). Increasing concentrations of heavy metals in the environment often leave a detectable trace in human and animal organisms (Bowen 1979; Maňkovská 1980; Holm and Wester 1988; Pokorny et al. 2004a, b).

Game animals, such as the European roe deer (*Capreolus capreolus*), have long been used as indicators of environmental contamination (Sawicka-Kapusta 1979; Maňkovská et al. 2012). Roe deer are found in high abundance across almost all of Europe and the Middle East. Roe deer show large behavioural plasticity and can occupy wide range of habitats, including those strongly affected by human activities (Burbaitė and Csanyi 2009). Despite broad geographical distribution, individual roe deer typically have small home ranges of 16–80 ha (Jeppesen 1990; Pandini and Cesaris 1997). Thus, it seems likely that concentrations of trace elements in roe deer tissues could reflect concentrations of pollutants in the local environment (Kucharczak et al. 2006). However, different trace elements might be deposited in different tissues at a varying rate (Ericson et al. 1991; Komarnicki 2000; Garcia et al. 2011; Kubaszewski et al. 2014). Also, there might be large age-related variation in the trace element content in different types of tissues (Kierdorf et al. 1989; Gasparik et al. 2004; Bilandžić et al. 2009; Rudy 2010; Garcia et al. 2011; Jarzyńska and Falandysz 2011; Lanocha et al. 2012).

Ecotoxicological studies of roe deer have mostly focused on heavy metal concentrations in blood (Baroni et al. 2000; Žele and Vengušt 2012; Humann-Ziehank et al. 2008) and soft tissues, such as liver, kidney, and muscle (Pokorny 2000; Pokorny and Ribarič-Lasnik 2002; Baloš et al. 2015; Durkalec et al. 2015; Lehel et al. 2016). This is understandable, because venison is allowed for consumption within the European Union (EU) and must comply with the EU standards (Ziembińska and Krasnowska 2007; Lehel et al. 2016). Trace element concentration was also studied in deer hair (Kucharczak et al. 2003, 2004, 2006) and feces (Babińska-Werka and Czarnowska 1988; Pokorny et al. 2004a, b). Finally, extensive ecotoxicological research has been performed on deer antlers (Sawicka-Kapusta 1979; Tataruch 1995; Kierdorf and Kierdorf 2000, 2001, 2002, 2003; Pokorny 2006; Pokorny et al. 2009; Jabłońska et al. 2016), possibly due to wide availability of hunting trophies. Other hard tissues, such as skeletal bones and teeth, have been much less researched (Maňkovská 1980; Zaccaroni et al. 2008; Sobota et al. 2011; Maňkovská et al. 2012). It remains unknown whether concentrations of trace elements in tissues change over the life cycle of roe deer and whether these age-related trends, if present, are similar for different types of tissues. We believe that such knowledge is crucial to draw reliable conclusions about the exposure of individuals or populations to environmental pollution while investigating concentrations of trace elements in animal tissues.

The purpose of this study was to measure concentrations of seven trace metals (barium, copper, iron, lead, manganese, strontium, zinc) and fluoride in skeletal bone and teeth of roe deer and to determine if they show significant variation with individual age. For this purpose, we collected permanent molars and fragments of mandible bone from more than 130 female roe deer in Central Poland. Although we had no quantitative data on environmental pollution in our sampling area, the trace elements chosen for this study are all toxic and potentially toxic, and they are among those in greatest commercial use or emission, likely exerting ecotoxicological effects on humans and wildlife (Wong et al. 2006).

## Materials and Methods

### Study Area

All samples were collected in Łódź voivodship, Central Poland. In terms of physiography, Łódź voivodship is situated on the border of two major units: the Central European Lowland and the Polish Highlands (Kondracki 2002). The entire study area has the lowest share of forests (21.1%) in the country. The rate of urbanization is 63.8%, and the share of agricultural area is 60.4%. Samples were collected in seven Game Breeding Centres (GBCs): Brzeziny (51°45′ N, 19°43′ E), Kolumna (51°34′ N, 19°13′ E), Kutno (52°14′ N, 19°08′ E), Poddębice (51°54′ N, 18°53′ E), Smardzewice (51°26′ N, 19°60′ E), Spała (51°31′ N, 20°11′ E), and Wieluń (51°11′ N, 18°44′ E), all managed by the Regional Directorate of State Forest in Łódź. All GBCs were located relatively close (25–100 km) to a large urban centre, Łódź (51°46′ N, 19°28′ E; 293 km^2^, 708,500 inhabitants).

### Sample Collection and Processing

Female roe deer were culled during regular hunting period from 30 September to 15 January in accordance with local hunting plans and regulations. A total sample consisted of 132 female skulls obtained in 2009–2014. Skull preparation followed standard procedures: boiling in water for 2–2.5 h, cleaning from soft tissues, rinsing in clean water, bleaching in oxidized water, and air drying for 24 h. Age of sampled specimens was evaluated based on dental wear (Przybylski 2008) by the members of the Regional Commission for Hunting Evaluation in Łódź. Tooth wear forms the mechanistic basis of senescence in ungulates (Gaillard et al. 1993) and have been frequently used for age determination in many cervid species (Brown and Chapman 1991; Ericsson and Wallin 2001; Høye 2006). Although tooth wear in roe deer has been reported to show some interpopulation variation due to differences in diet and habitat (Hewison et al. 1999), our samples were collected within small geographical area, which was characterized by relatively uniform environmental conditions. Age of roe deer in our sample varied between 2 and 12 years. For the purpose of analyses, animals were grouped into four age classes: (1) 2 years old (*n* = 49), (2) 3–4 years old (*n* = 35), (3) 5–6 years old (*n* = 27), and (4) > 6 years old (*n* = 19). The third permanent molar and a small fragment of mandible bone were collected from each skull and used in further analyses. The teeth were usually collected from the left side, but in a few cases teeth from the right side were extracted, because those on the left side were mechanically damaged, missing, or exhibited pathological alterations.

### Measurements of Trace Metal and Fluoride Concentrations

All teeth and bone fragments were rinsed in deionized water to remove externally absorbed elements. The samples used for the measurements of metal concentrations were dried in an oven at 70 °C for 48 h and then weighed to the nearest 0.01 g. Average mass of dried samples was 0.71 ± 0.02 [SE] g and 0.48 ± 0.01 [SE] g for teeth and bone, respectively. Dried samples were dissolved in the proportion of 1:15 in 65% nitric acid, kept in 20 °C for 24 h, and then digested at 105 °C for another 24 h using a graphite digestion block (DigiPREP Mini, SCP Science, Quebec, Canada). After digestion, all samples were diluted with deionized water to the total volume of 30 mL and stored in polypropylene metal-free vials at 20 °C until analysis.

Concentrations of barium, copper, iron, lead, manganese, strontium, and zinc were measured in the samples using atomic absorption spectrophotometer SpectrAA 300A AAS, GTA-96 graphite tube atomizer, and programmable sample dispenser (Varian Techtron, Melbourne, Australia). The analyses were performed in the Laboratory of Computer and Analytic Techniques, Faculty of Biology and Environmental Protection, University of Łódź. For copper, iron, lead, manganese, and zinc, we used certified reference material (ERM-186 pig kidney) from the Institute for Reference Materials and Measurements (Geel, Belgium). For strontium, we used Strontium Standard for AAS (TraceCERT^®^, 1000 mg/L Sr in nitric acid), and for barium, we used Barium Standard for AAS (TraceCERT^®^, 1000 mg/L Ba in nitric acid) to verify the quality and accuracy of the analyses. Recovery rates for the certified reference materials were within an acceptable margin.

Measurements of fluoride concentration followed methodology recommended by Campus et al. (2007). First, we powdered 1.2 g of each tooth and bone sample in a ball mill Mixer Mill MM 400 (Retsch, Germany) with zirconium oxide beads (frequency 25 Hz, time 60 s). Powdered samples were transferred to a volumetric flask, dissolved in 8 mL of 37% HCl solution, and then diluted with deionized water to the total volume of 10 mL. A 5-mL aliquot of the above solution was transferred to another volumetric flask, diluted 1:1 with deionized water, neutralized with a 6 M NaOH solution to pH 4.5, and diluted with deionized water to the total volume of 25 mL. Sample solution was diluted with TISAB (1:1) and fluoride concentration was measured with ion-selective fluoride electrode (Hydromet S.C., Gliwice, Poland). All trace element concentrations were expressed in mg per kg dry mass.

### Statistical Analyses

Before analyses, we identified outliers using criteria of > 4 SD. Outlier analyses were conducted separately for teeth and bone samples. Between one and three outliers were identified in 10 of 16 analysed measurements, whereas 6 measurements showed no outliers (Fig. S1 in the Electronic Supplementary Material). All outliers were removed from the dataset. After outlier removal, measurements with high (> 1) skewness (copper, iron, lead, manganese, and fluoride) were log-transformed to improve normality.

The effects of age and sample type (teeth vs. bone) on trace metal and fluoride concentrations were analysed with general linear mixed models (GLMMs). Because teeth and bone samples were collected from the same individuals, we included individual identity as a random factor to avoid pseudoreplication (Hurlbert 1984). The effect of year also was included as a random factor to control for interannual variation in the collected measurements. Age and sample type were included as fixed factors. To test whether age-related differences were similar for both sample types, we entered age-sample interaction in each model. GLMM models were fitted using the restricted maximum likelihood (REML) method. With this approach, denominator degrees of freedom are calculated by using a Satterthwaite approximation, which can result in fractional degrees of freedom (Satterthwaite 1946). The results of full models were reported. For measurements that showed significant age-related variation, we used contrast analysis (following recommendations by Ruxton and Beauchamp 2008) to test for an a priori hypothesis of linear increase or decrease with age. We also used Tukey post hoc comparisons to test for pairwise differences between age classes. All statistical analyses were conducted in JMP 12.1.0 (SAS Institute Inc., Cary, NC).

## Results

We found that mean concentrations of five trace metals (copper, lead, manganese, strontium, and zinc) differed significantly between teeth and bone (Tables 1, 2). Specifically, concentrations of copper and lead were higher in bone, while concentrations of manganese, strontium, and zinc were higher in teeth (Tables 1, 2). No differences between teeth and bone were recorded in the concentration of barium, iron, and fluoride (Tables 1, 2).

**Table 1:** Mean (±SE) concentrations of seven trace metals and fluoride in bone (mandible) and teeth (third permanent molar) in roe deer from Central Poland

Trace element	Bone		Teeth	
	Mean ± SE	*n*	Mean ± SE	*n*
Barium	209.25 ± 6.12	131	200.74 ± 6.39	132
Copper	5.74 ± 0.40	130	5.28 ± 0.27	130
Iron	21.71 ± 0.61	130	20.68 ± 0.49	132
Lead	0.62 ± 0.04	131	0.51 ± 0.04	130
Manganese	6.33 ± 0.18	132	82.78 ± 6.70	131
Strontium	89.09 ± 1.98	132	92.03 ± 2.29	132
Zinc	94.52 ± 1.19	130	107.49 ± 2.10	132
Fluoride	4.82 ± 0.45	129	3.81 ± 0.38	130

All concentrations are given in mg per kg dry mass

**Table 2:** Effects of age and sample type (bone vs. tooth) on the concentrations of seven trace metals and fluoride

Factor	Barium		Copper		Iron		Lead	
	*F*	*p*	*F*	*p*	*F*	*p*	*F*	*p*
Age	7.70	0.065	0.68	0.57	**6.01**	**<** **0.001**	1.23	0.30
Sample type	0.44	0.51	**5.16**	**0.025**	0.89	0.35	**15.46**	**<** **0.001**
Age*Sample type	**10.19**	**<** **0.001**	0.37	0.77	0.73	0.53	0.69	0.56

	Manganese		Strontium		Zinc		Fluoride	
	*F*	*p*	*W*	*p*	*F*	*p*	*F*	*p*
Age	**2.99**	**0.033**	0.77	0.51	**13.63**	**<** **0.001**	**4.31**	**0.006**
Sample type	**1105.3**	**<** **0.001**	**7.35**	**0.008**	**97.41**	**<** **0.001**	2.80	0.097
Age*Sample type	**4.74**	**0.004**	**2.77**	**0.045**	**20.87**	**<** **0.001**	2.25	0.086

Significant terms are marked in bold

**Fig. 1:**
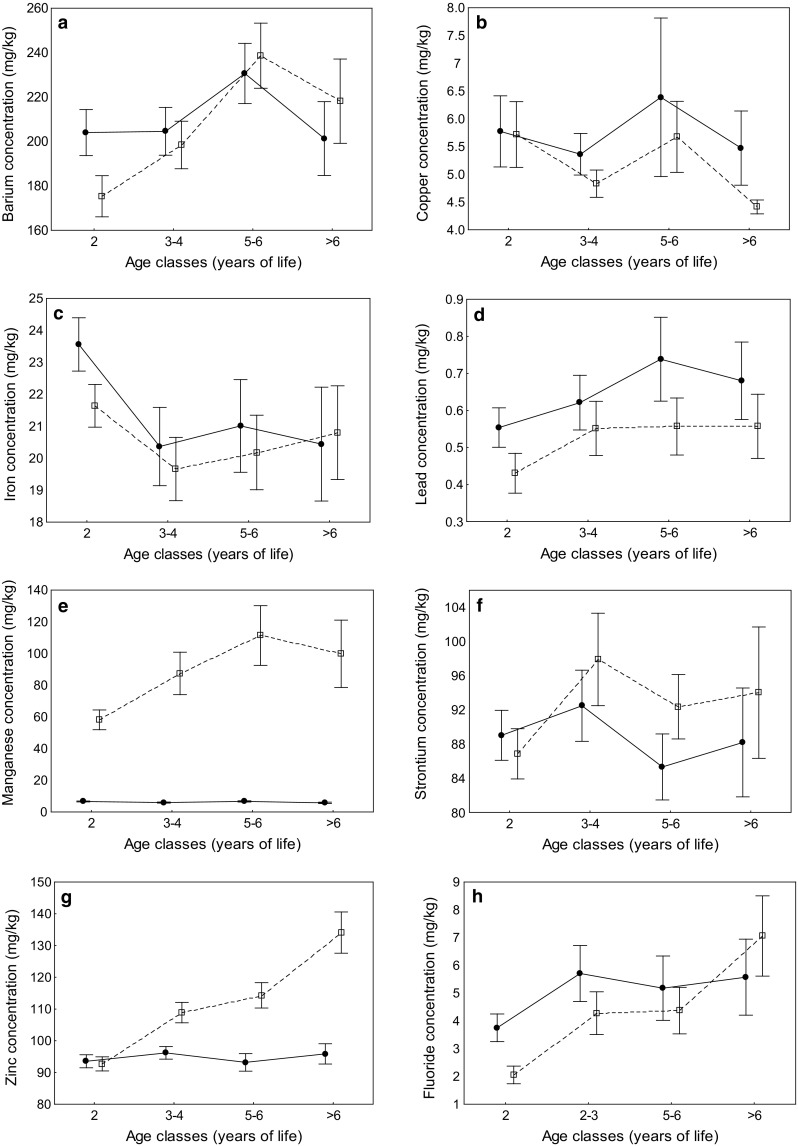
Age-related variation in the concentrations of seven trace metals (**a** barium, **b** copper, **c** iron, **d** lead, **e** manganese, **f** strontium, **g** zinc) and fluoride (**h**) in bone (solid line, line, filled circles) and teeth (dotted line, open squares) of roe deer. Means ± SE are presented

Significant age variation was found in the concentrations of five of seven trace metals (barium, iron, manganese, strontium, and zinc), although in most cases the effect of age depended on the sample type (Table 2). Concentration of only one trace metal, iron, varied with age irrespectively of sample type. Concentration of iron in both teeth and bone showed a decreasing linear trend with age (contrast analysis: *F*_1,122.5_ = 8.99, *p* = 0.003), but the only significant difference was between youngest individuals (2 years old) and older age classes (Tukey: all *p* < 0.05; Fig. 1c). The effect of age on all other trace metal concentrations depended on the type of sample, as indicated by significant age-sample interactions (Table 2). In all of these cases, we recorded no age-related variation in trace metal concentrations in bone (barium: *F*_3,126.3_ = 0.99, *p* = 0.40; manganese: *F*_3,124.9_ = 1.56, *p* = 0.20; strontium: *F*_3,126.4_ = 1.16, *p* = 0.33; zinc: *F*_3,122.3_ = 0.70, *p* = 0.55; Fig. 1). In contrast, teeth samples showed significant age-related variation in the concentrations of barium (*F*_3,125.3_ = 4.75, *p* = 0.004; Fig. 1a), manganese (*F*_3,125.4_ = 4.85, *p* = 0.003; Fig. 1e), and zinc (*F*_3,126.2_ = 23.31, *p* < 0.001; Fig. 1g), whereas no age variation was found for strontium concentration (*F*_3,126.9_ = 1.03, *p* = 0.38; Fig. 1f). Concentrations of barium, manganese, and zinc in teeth showed significant linear increase with age, as indicated by the contrast analysis (barium: *F*_1,126.2_ = 8.78, *p* = 0.004; manganese: *F*_1,125.4_ = 7.70, *p* = 0.006; zinc: *F*_1,126.2_ = 57.35, *p* < 0.001). An interaction between age and sample type for fluoride concentration approached significance (Table 2). Analysis of fluoride concentrations separately for the two types of samples revealed no age-related differences in bone (*F*_1,122_ = 0.97, *p* = 0.33) and a significant increase with age in teeth (contrast analysis: *F*_1,120.4_ = 13.40, *p* < 0.001; Fig. 1h). No age-related differences were recorded for copper and lead (Table 2; Fig. 1b, d).

## Discussion

Our study provided strong evidence for age-related variation in the concentrations of several trace elements in permanent teeth of roe deer from Central Poland. Concentrations of three trace metals (barium, manganese, and zinc) in teeth of deer showed positive linear relationships with animal’s age, indicating that some elements can accumulate in tooth structure throughout life. A similar trend was observed for fluoride concentration in teeth. In contrast, none of trace elements in bone showed an age-related increase in concentration, suggesting that bone and teeth show different patterns of trace element accumulation.

Differences in age-related patterns of bioaccumulation between bone and teeth can be most likely attributed to much higher turnover rate of bone when compared with tooth structure (Malara et al. 2016). Bone tissue, mainly formed from carbonated hydroxyapatite, is remodelled throughout entire animal life and its microelements can be transported to other tissues or excreted from organism. Specifically, bone can serve as a metal reservoir, because trace metals accumulated in this tissue are released into the bloodstream during its reconstruction. We are not aware of any quantitative data on bone turnover rate in the roe deer. However, it is estimated that more than 10% of total human bone tissue is remodelled each year, making an adult skeleton completely rebuilt in less than 10 years (Arnett and Henderson 1998). In animals with shorter lifespan, such as the roe deer (maximum recorded lifespan of 17.5 years according to the AnAge database; De Magalhaes and Costa 2009), the period of the total skeletal turnover might be even shorter. Bone tissue is characterized by longer redevelopment period than most soft tissues, and thus, its trace element content is thought to reflect long-term exposure to environmental pollution (Glimcher 2006; Zaichick et al. 2011; Malara et al. 2016). Nevertheless, our study of roe deer suggests that, at least in this species, bone might not be a reliable indicator of throughout-life exposure to contaminants.

This is in sharp contrast to our findings for permanent teeth. Although ungulates have two generations of teeth, the deciduous set of teeth is replaced relatively early in the postnatal development and permanent teeth usually start to erupt before individuals finish their first year of life (Loe et al. 2004). When permanent teeth are developed their structure does not undergo remodelling and they are likely to bioaccumulate trace elements effectively from food and air. Consequently, long-term exposure of an organism to certain pollutants is likely to produce positive relationships between trace element concentrations in permanent teeth and individual age. Although we are aware that trace element content of permanent teeth cannot capture an exposure to pollutants in early postnatal period (first year of life), we suggest that permanent teeth are likely to more reliably, compared with bone tissue, indicate contamination that occurs throughout the entire adult life of roe deer. High reliability of teeth as the marker of environmental pollution by heavy metals also has been reported for small mammals, e.g., the bank vole (*Clethrionomys glareolus*) (Appleton et al. 2000).

Information on age-related variation in trace element concentrations in roe deer and other wild-living mammals is scarce and fragmentary. Many authors investigated trace elements in antlers, bone, and soft tissues of roe deer, but they either neglected age of animals in their studies (Sileo and Beyer 1985; Babińska-Werka and Czarnowska 1988; Zaccaroni et al. 2008; Baloš et al. 2015; Durkalec et al. 2015) or they distinguished two broad age categories of young and adults (Nowicka et al. 2006; Millán et al. 2008; Sobota et al. 2011). For example, Garcia et al. (2011) tested for differences in the contamination of liver, kidney, and muscle by cadmium, lead, and zinc between young (< 3 years old) and adult roe deer. Age-related differences were found for all tissues; cadmium and lead concentrations were higher in adults, whereas zinc concentration was higher in young individuals (Garcia et al. 2011). While separation of age into the categories of young and adults can provide some insight into age variation in trace element content, it is certainly insufficient to draw any detailed conclusions on how trace element concentrations change during an adult life. In contrast, our classification of age into four relatively narrow categories allowed us to effectively explore age-related trends in trace element bioaccumulation by roe deer.

In our study, age-related accumulation of trace elements in permanent teeth has been recorded for barium, manganese, and zinc. Consistently, mean concentrations of manganese and zinc were significantly higher in teeth than in bone tissue, and the same pattern was found for strontium. In fact, all these elements are thought to be important for hard tissues, especially for enamel (Lynch 2011) but possibly also for bone. For example, Marie et al. (2001) showed that application of large strontium dose had a positive effect on the hardness of the bone tissue in humans. However, two other trace elements, copper and lead, showed the opposite trends, as higher concentrations were recorded in bone that in teeth. Similar results were found in the forest reindeer (*Rangifer tarandus fennica*) from Karelia, Russia, where lead concentration was higher in bone than in teeth and antler (Medvedev 1995). In our study, neither copper nor lead showed any age-related variation in bone or teeth samples. Consistent with these results, an analysis of antlers and bone tissue by Sobota et al. (2011) provided no support for differences in lead concentration between young and adult roe deer. In contrast, Kierdorf and Kierforf (2002) found that lead concentration in antlers of young roe deer was significantly lower than in older individuals. Apparent inconsistency between these two studies suggests that age-related patterns in trace element content may vary between populations, possibly as a result of spatial and temporal variation in environmental pollution. In fact, trace element concentrations in roe deer have been reported to depend on the soil and water contamination and to show large geographical variation (Anderson et al. 2011). Accumulation rate of trace elements is also acknowledged to depend on the physiological state of animals (Zakrzewska et al. 2005), which may show some interpopulation variation. Finally, iron was the only element in our study of roe deer that had higher concentration in young than adult individuals, both in teeth and bone. While the mechanism responsible for this pattern merits further research, similar trends were found in other wild-living mammals, such as red fox *Vulpes vulpes* (Lanocha et al. 2012) and domestic mouse *Mus musculus* (Kennedy et al. 1986).

## Conclusions

Our study revealed striking differences in age-related patterns of trace element bioaccumulation between bone and permanent teeth of roe deer. Most importantly, we found that concentrations of several trace elements in teeth increased with individual age, whereas no such trends were recorded for bone tissue. The results suggest that permanent teeth are likely to indicate reliably throughout-life intoxication by environmental pollution in the roe deer and possibly in other mammal species. Our study also reinforces the need to account carefully for age-related variation in ecotoxicological research on wild-living animals.

## Electronic supplementary material

Below is the link to the electronic supplementary material.

Supplementary material 1 (PDF 58 kb)

Supplementary material 2 (XLSX 27 kb)
