# Supplementary material for: Age-Related Patterns in Trace Element Content Vary Between Bone and Teeth of the European Roe Deer (Capreolus capreolus)
Source: Arch Environ Contam Toxicol. 2017 Oct 25;74(2):330–8. doi: 10.1007/s00244-017-0470-1 (PMC5807465; doi:10.1007/s00244-017-0470-1)
Supplement: Supplementary file 1 — Supplementary material 1 (PDF 58 kb) [file 244_2017_470_MOESM1_ESM.pdf]

Electronic Supplementary Material

Age-related patterns in trace element content vary between bone and teeth of the European roe deer (*Capreolus capreolus)*

Jan Demesko[1] · Janusz Markowski[1] · Mirosława Słaba[2] · Janusz Hejduk[1] · Piotr Minias[1]

[1] Department of Biodiversity Studies and Bioeducation, Faculty of Biology and Environmental Protection, University of Łódź, Banacha 1/3, 90-237 Łódź, Poland

[2] Department of Industrial Microbiology and Biotechnology, Faculty of Biology and Environmental Protection, University of Łódź, Banacha 12/16, 90-237 Łódź, Poland

Corresponding author: Jan Demesko, e-mail: jan.demesko@biol.uni.lodz.pl

Fig. S1 Histograms for concentrations of seven trace metals and fluoride in bone and teeth of roe deer. Outliers are marked with arrows

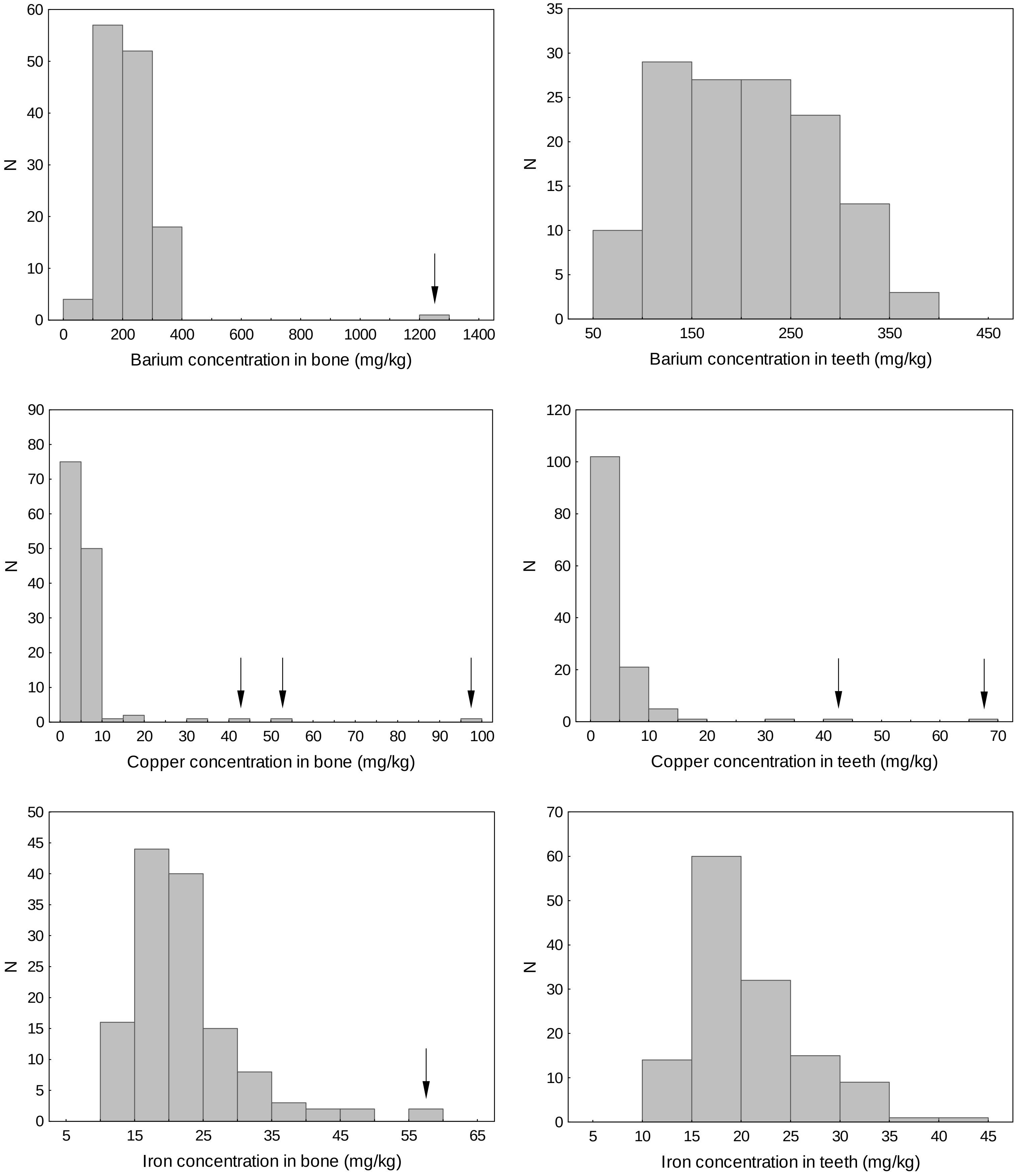

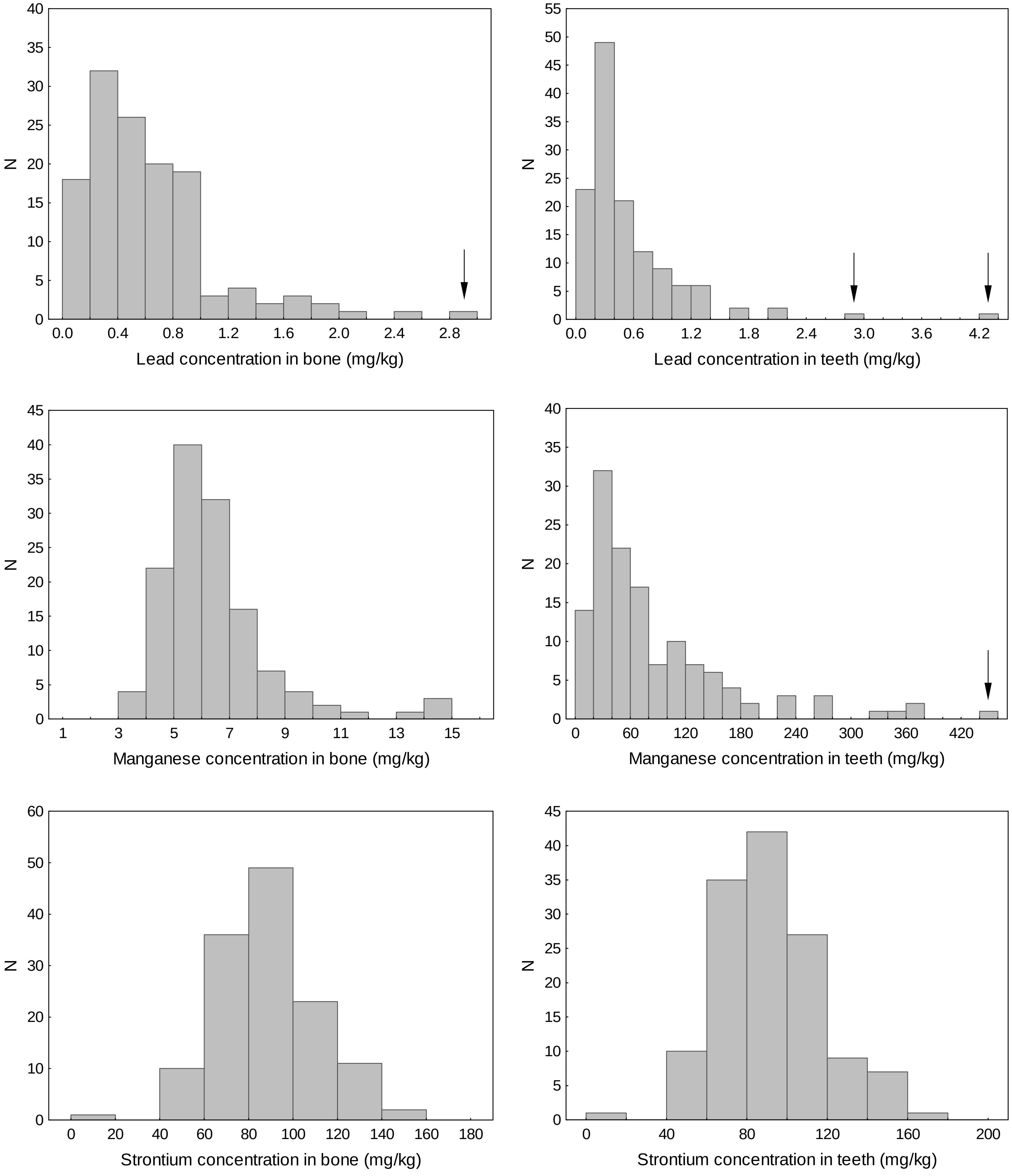

N
Lead concentration in bone (mg/kg)
0.0 0.4 0.8 1.2 1.6 2.0 2.4 2.8
0 5 10 15 20 25 30 35 40
N
Lead concentration in teeth (mg/kg)
0.0 0.6 1.2 1.8 2.4 3.0 3.6 4.2
0 5 10 15 20 25 30 35 40 45 50 55
N
Manganese concentration in bone (mg/kg)
1 3 5 7 9 11 13 15
0 5 10 15 20 25 30 35 40 45
N
Manganese concentration in teeth (mg/kg)
0 60 120 180 240 300 360 420
0 5 10 15 20 25 30 35 40
N
Strontium concentration in bone (mg/kg)
0 20 40 60 80 100 120 140 160 180
0 10 20 30 40 50 60
N
Strontium concentration in teeth (mg/kg)
0 40 80 120 160 200
0 5 10 15 20 25 30 35 40 45

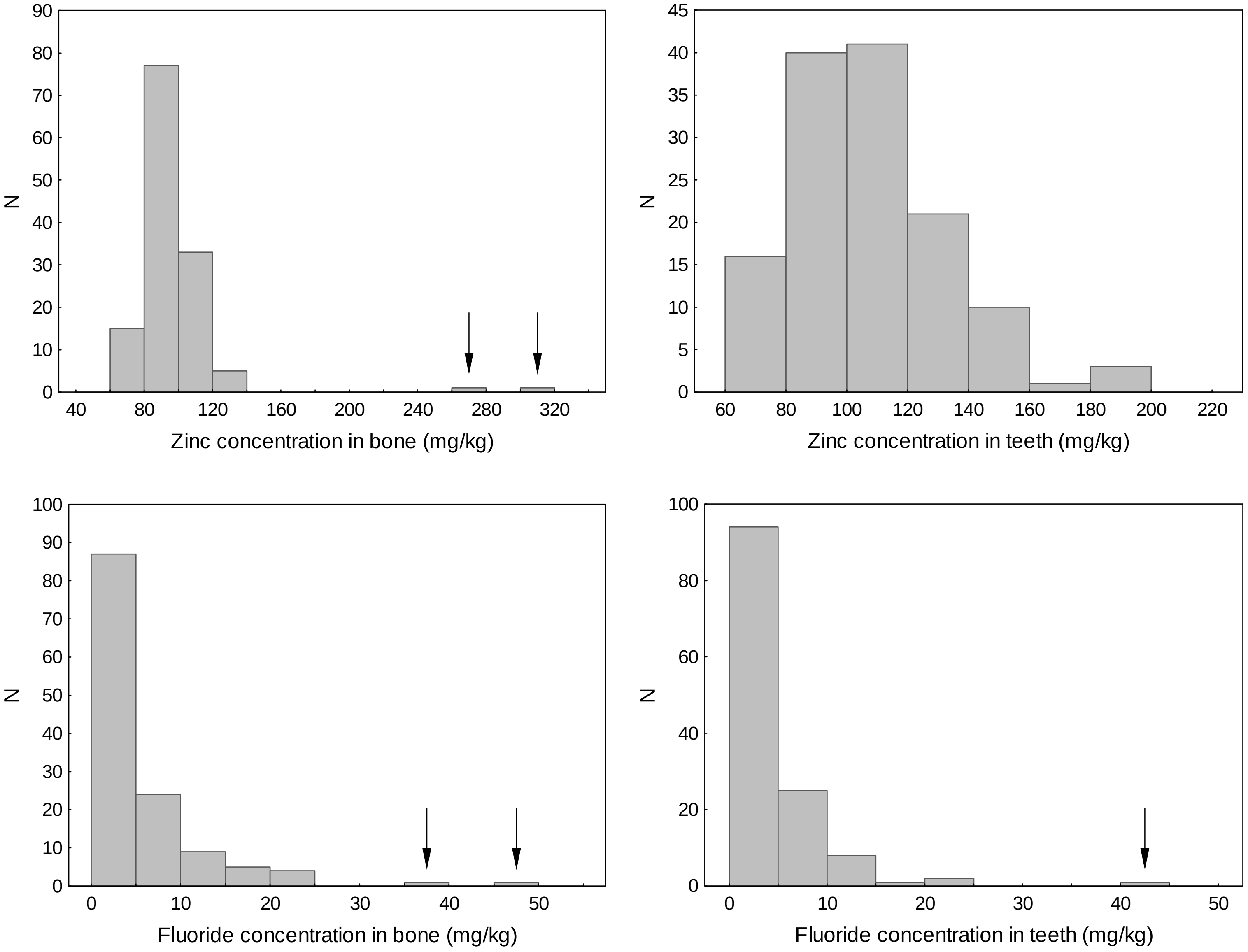

N
Zinc concentration in bone (mg/kg)
N
Zinc concentration in teeth (mg/kg)
N
Fluoride concentration in bone (mg/kg)
N
Fluoride concentration in teeth (mg/kg)
